# Human Blood CD1c^+^ Dendritic Cells Promote Th1 and Th17 Effector Function in Memory CD4^+^ T Cells

**DOI:** 10.3389/fimmu.2017.00971

**Published:** 2017-08-17

**Authors:** Ingrid M. Leal Rojas, Wai-Hong Mok, Frances E. Pearson, Yoshihito Minoda, Tony J. Kenna, Ross T. Barnard, Kristen J. Radford

**Affiliations:** ^1^Cancer Immunotherapies Laboratory, Mater Research Institute, The University of Queensland, Translational Research Institute, Woolloongabba, QLD, Australia; ^2^Institute of Health and Biomedical Innovation, Queensland University of Technology, Translational Research Institute, Woolloongabba, QLD, Australia; ^3^School of Chemistry and Molecular Biosciences, The University of Queensland, St Lucia, QLD, Australia

**Keywords:** dendritic cell, CD141^+^ dendritic cells, CD1c^+^ dendritic cells, Th1, Th17, memory CD4^+^ T cells, toll-like-receptor

## Abstract

Dendritic cells (DC) initiate the differentiation of CD4^+^ helper T cells into effector cells including Th1 and Th17 responses that play an important role in inflammation and autoimmune disease pathogenesis. In mice, Th1 and Th17 responses are regulated by different conventional (c) DC subsets, with cDC1 being the main producers of IL-12p70 and inducers of Th1 responses, while cDC2 produce IL-23 to promote Th17 responses. The role that human DC subsets play in memory CD4^+^ T cell activation is not known. This study investigated production of Th1 promoting cytokine IL-12p70, and Th17 promoting cytokines, IL-1β, IL-6, and IL-23, by human blood monocytes, CD1c^+^ DC, CD141^+^ DC, and plasmacytoid DC and examined their ability to induce Th1 and Th17 responses in memory CD4^+^ T cells. Human CD1c^+^ DC produced IL-12p70, IL-1β, IL-6, and IL-23 in response to R848 combined with LPS or poly I:C. CD141^+^ DC were also capable of producing IL-12p70 and IL-23 but were not as proficient as CD1c^+^ DC. Activated CD1c^+^ DC were endowed with the capacity to promote both Th1 and Th17 effector function in memory CD4^+^ T cells, characterized by high production of interferon-γ, IL-17A, IL-17F, IL-21, and IL-22. These findings support a role for CD1c^+^ DC in autoimmune inflammation where Th1/Th17 responses play an important role in disease pathogenesis.

## Introduction

Dendritic cells (DC) are a heterogeneous population of leukocytes with specialized subsets responsible for driving specific immune responses (1–3). They can be categorized into four major subtypes that are largely conserved across different tissues and species; MoDC that differentiate from monocytes under inflammatory conditions, plasmacytoid DC (pDC) that are the major producers of type I interferons (IFN), and conventional DC (cDC), which are derived from distinct committed DC precursors and are further subdivided into cDC1 and cDC2 subsets (1, 2). DC subsets differentially express a suite of pattern recognition receptors (PRRs), including the toll-like-receptors (TLRs), C-type lectin receptors (CLRs), NOD-like receptors (NLRs), and RIG-I receptors that they use to sense pathogens and damage to the host. Specific types of immune responses are directed by individual DC subsets and the different cytokines and IFN they produce as a result of PRR ligation.

The cDC1 subset comprises, in the mouse, lymphoid tissue resident CD8^+^ DC and non-lymphoid tissue resident CD103^+^ DC, and in humans, the CD141 (BDCA3)^+^ DC (2, 3). cDC1 require FLT3L and transcription factors IRF8, ID2, and Batf3 for their development and share expression of chemokine receptor XCR1, the CLR Clec9A, nectin-like protein 2 (CADM1), and TLR3 across species. In mice, these DC are crucial for immunity to intracellular infections and cancer, owing to their ability to produce high levels of IL-12p70, induce T helper (Th)-1 responses, and cross-present exogenous antigen for priming of CD8^+^ cytolytic T cell responses (CTL) (1). The cDC2 subset of cDC in mice is commonly referred to as CD11b^+^ DC in the murine lymphoid and non-lymphoid tissues, and in humans as CD1c (BDCA1)^+^ DC (1–3). In mice, cDC2 are FLT3L and IRF4 dependent and share significant overlap in phenotype with cells of the monocyte lineage (1). Mouse cDC2 play a key role in the induction of immune responses to extracellular pathogens, owing to their ability to produce IL-23 that promotes Th17 type responses (4–8).

Although closely aligned at the level of gene expression (9, 10), the degree to which human DC subsets share similar functions with their mouse counterparts is less understood. Most functional studies on human DC have examined blood as the most accessible source of tissue and while these are closely related to DC in lymphoid tissues and strongly defined by ontogeny, the DC residing in the non-lymphoid tissues are also heavily influenced by the microenvironment (11). Like mouse cDC1, human blood CD141^+^ DC express high levels of TLR3 and are major producers of IFN-λ (12, 13). They also excel at cross-presentation of antigen from necrotic cells, similar to mouse cDC1 (12, 14, 15). Mouse cDC1 produce high levels of IL-12p70 but in humans this may be more dependent on tissue and stimuli since thymic CD141^+^ DC are the main DC subset producing IL-12 while blood CD141^+^ DC are not major producers of this cytokine (1, 3, 16). Human lung CD1c^+^ DC produce IL-23 and promote Th17 responses following exposure to fungal stimuli (7) and blood CD1c^+^ DC activated *via* TLR2 also induce Th17 responses (17), suggesting involvement of this lineage in immunity to extracellular pathogens, similar to mouse cDC2 (7, 17). However, human CD1c^+^ DC also secrete high levels of IL-12p70, particularly when stimulated with the TLR7/8 ligand, R848, coupled with TLR3 or 4 ligands, or mycobacteria (17–20). Several studies have also shown that CD1c^+^ DC in blood and lymphoid tissue can cross-present soluble antigen for recognition by CD8^+^ CTL (12, 20–22). Therefore, human CD1c^+^ DC may possess the capacity to induce both Th1 and Th17 responses in some situations and this would represent a key interspecies difference in DC function.

The stimuli for the differentiation of naïve human Th1 and Th17 CD4^+^ T cells are tightly regulated and differ from the conditions needed for maintenance and effector function of memory CD4^+^ T cells. Differentiation of human Th17 cells from naïve CD4^+^ T cells requires IL-1β and IL-6 (17) while effector function of memory CD4^+^ Th17 cells is dependent on IL-1β and IL-23 (23, 24). Priming of naïve CD4^+^ T cells in the presence of both IL-12p70 and IL-23 results in a Th1 response as IL-12p70 inhibits Th17 differentiation (17, 25). However, IL-12p70 and IL-23 together can drive effector function in memory CD4^+^ cells and this underpins the pathogenesis of many human inflammatory conditions (26). IL-23 drives effector function of Th17 cells characterized by production of IL-17A, IL-17F, IL-21, and IL-22 (26) and the presence of IL-12 reprograms memory Th17 cells into a “Th17/Th1” phenotype that are more aggressive and pathogenic, favoring autoimmune disease progression (11, 27–30).

The role of specific human DC subsets in driving memory CD4^+^ T cell responses is presently unclear. In this study, we therefore examined the combination of TLR ligands that induce human blood DC subsets to produce both Th1 and Th17 polarizing cytokines and examined the capacity of TLR-activated DC to promote memory Th1 and Th17 responses. Our data show that CD1c^+^ DC, but not CD141^+^ DC, pDC, or monocytes, produce IL-1, IL-6, IL-12, and IL-23 simultaneously in response to R848 (a TLR 7/8 ligand) combined with LPS (a TLR 4 ligand) or poly I:C (an activator of TLR3 or RIG-I and MDA-5), and promote Th1 and Th17 effector function in memory CD4^+^ T cells.

## Materials and Methods

### DC Isolation and Culture

Whole blood and leukapheresis products from healthy volunteers were obtained for this study following approval from Mater Health Services Human Research Ethics Committee and with written informed consent. Peripheral blood mononuclear cells were isolated by Ficoll-Paque Plus density gradient centrifugation (GE Healthcare). For experiments comparing CD1c^+^ DC and CD14^+^ monocytes, CD1c^+^ DC were isolated to >85% purity using the human BDCA1 DC isolation kit and monocytes isolated to >98% purity using the human CD14 isolation kit (Miltenyi Biotec) according to the manufacturer’s instructions. For comparisons of human DC subsets, DC were first enriched using a pan DC enrichment kit (Stemcell Technologies), then labeled with fluorescently conjugated mouse anti-human Abs specific for CD3 (OKT3), CD14 (HCD14), CD16 (3G8), CD19 (HIB19), CD20 (2H7), CD56 (HCD56), CD1c (L161), CD141 (M80), CD123 (6H6), HLA-DR (L243), and Live/Dead Aqua Dye (all from Biolegend). Cells were sorted using a MoFlow^®^ Astrios™ (Beckman Coulter) and human DC identified as live singlet cells that were lineage (CD3, CD14, CD16, CD19, CD20, CD56)^−^ HLA-DR^+^ then further segregated by expression of CD141, CD1c, and CD123 (for pDC) (Figure S1 in Supplementary Material).

### Stimulation with TLR Ligands

Sorted DCs and monocytes were cultured in RPMI 1640 medium, supplemented with 10% FCS, 100 U/ml penicillin, 100 µg/ml streptomycin, 2 mM L-glutamine, 1 mM sodium pyruvate, 0.1 mM non-essential amino acids, 10 mM HEPES buffer solution (all obtained from Invitrogen), and 50 µM 2-mercaptoethanol (Sigma-Aldrich) at a density of 50,000 per 50 μl in 96 well V-bottom plates. Cells were stimulated with 10 ng/ml LPS (Sigma Aldrich), 1 μg/ml R848 (Invivogen), 25 μg/ml poly I:C (Invivogen), or 1 µM CpG ODN 2216 (Miltenyi Biotec) alone or in various combinations for 20 h at 37^o^C, 5% CO_2_. Supernatants were harvested and cytokines measured using ELISA kits for IL-1β, IL-23, IFN-λ (R&D Systems), IL-6, IL-12p70 (BD Biosciences), and IFN-α (Interferon Source) or by flow cytometry using a LegendPlex kit (Biolegend) that was acquired on an LSR Fortessa X20 (BD Biosciences) and analyzed using VigeneTech software Version 7.0.

### Polarization of Memory CD4^+^ T Cells

Memory CD4^+^ T cells were isolated from PBMCs to >95% purity (CD4^+^ CD45RO^+^ CD45RA^−^) using a human memory CD4^+^ T cell Enrichment Kit (StemCell Technologies) according to manufacturer’s instructions. Human monocytes or DC untreated or activated with R848 and LPS or poly I:C were collected and washed with PBS, then 10,000 cells were co-cultured with 100,000 autologous memory CD4^+^ T cells in 100 μl culture medium containing 25 ng/ml IL-7 and 25 ng/ml IL-15 in 96-well V-bottom plates for 7 days (31). Supernatants were collected and cytokines IL-2, IL-4, IL-5, IL-6, IL-9, IL-10, IL-13, IL-17A, IL-17F, IL-21, IL-22, IFN-γ, and TNF-α were measured by flow cytometry on an LSR Fortessa X20 using a Human T helper LegendPlex kit (Biolegend) analyzed using VigeneTech software Version 7.0. T cell proliferation and intracellular staining for IL-17A and IFN-γ was assayed by first labeling memory CD4^+^ T cells with carboxyfluorescein succinimidyl ester (Molecular Probes) prior to co-culture with DC or monocytes. After 7 days cells were restimulated with phorbol 12-myristate 13-acetate (PMA) and ionomycin (Sigma Aldrich) for 6 h with Golgistop (BD Pharmingen) added for the final 2 h. Cells were fixed, permeabilized, and stained with anti-human IL-17A (eBio64DEC17) and IFN-γ (B27, BD Pharmingen) or matching isotype controls. Data were acquired on an LSR Fortessa X20 flow cytometer and analyzed using FlowJo Version 8.8.7 software.

### Transcriptome Analysis

Microarray expression data for human circulating blood DC and monocyte subsets was obtained from the GEO database GSE35457 (10). To examine changes in TLR gene expression following activation of human DC subsets *in vivo*, we used GEO dataset GSE99666. For this dataset, human CD141^+^ DC and CD1c^+^ DC were generated from human CD34^+^ progenitor cells following transfer into neonatal immunodeficient NSG-A2 mice (“humanized” mice). Global transcriptome, phenotype, and functional analyses have demonstrated that the CD141^+^ DC and CD1c^+^ DC that develop in this model closely resemble their human blood counterparts, making humanized mice a robust model for studying human DC *in vivo* (32). Briefly, humanized mice were treated intravenously with poly I:C or R848 and RNA extracted from purified DC subsets 2 h after activation. These experiments were approved by the University of Queensland Animal Ethics Committee. RNA was processed for hybridization and scanning using an Illumina HumanHT-12 v4 Expression system. Quality control, normalization, and log 2 transformation of the raw expression data were performed using the *Lumi* Bioconductor package (33) and integrated into the Stemformatics platform [www.stemformatics.org (34)] for visualization.

### Statistical Analysis

Data were log transformed and paired *t*-tests were used to compare a single condition between two cell subsets from the same donor. Data were considered statistically significant at *p* ≤ 0.05.

## Results

### CD1c^+^ DC Produce High Levels of IL-1β, IL-6, IL-12p70, and IL-23 in Response to R848 Combined with LPS or Poly I:C

**Figure 1:**
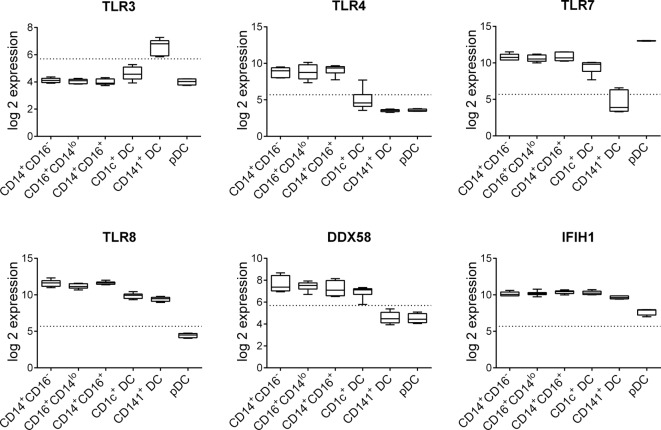
Pattern recognition receptor gene expression by human blood dendritic cells (DC) and monocyte subsets. Normalized log 2 gene expression was calculated from data for CD14^+^CD16^−^, CD16^+^CD14^lo^, and CD14^+^CD16^+^ human blood monocyte subsets as well as blood CD1c^+^ DC, CD141^+^ DC, and plasmacytoid DC obtained from GEO GSE35457 (10). Boxes represent 25th–75th percentiles ± minimum and maximum values with line at the median from four to six individual donors. Dotted line is the expression threshold determined by the bi-modal distribution (detected versus non-detected probes).

R848 is a ligand for TLR7 and TLR8, while LPS simulates TLR4 and poly I:C can activate *via* either TLR3 or RIG-I and MDA-5. Expression of these PRRs has previously been described in human DC and monocyte subsets but their expression levels have not been directly compared. Our analysis of publically available datasets (10) showed that human blood CD14^+^CD16^−^, CD16^+^CD14^lo^, and CD14^+^CD16^+^ monocyte subsets expressed similar high levels of *TLR4, TLR7, TLR8, DDX58* (RIG-I), and *IFIH1* (MDA-5) but not *TLR3* (Figure 1). Blood CD1c^+^ DC expressed similar or slightly lower levels of *TLR7, TLR8, DDX58*, and *IFIH1*compared with monocytes and weakly expressed *TLR3* and *TLR4* (Figure 1). CD141^+^ DC expressed the highest levels of *TLR3* and comparable levels of *TLR8* and *IFIH1* to CD1c^+^ DC while *TLR4, TLR7*, and *DDX58* were below the detection threshold. pDC expressed the highest levels of *TLR7* and weakly expressed *IFIH1* while *TLR3, TLR4, TLR8, DDX58*, and *IFIH1* were below the detection threshold (Figure 1).

**Figure 2:**
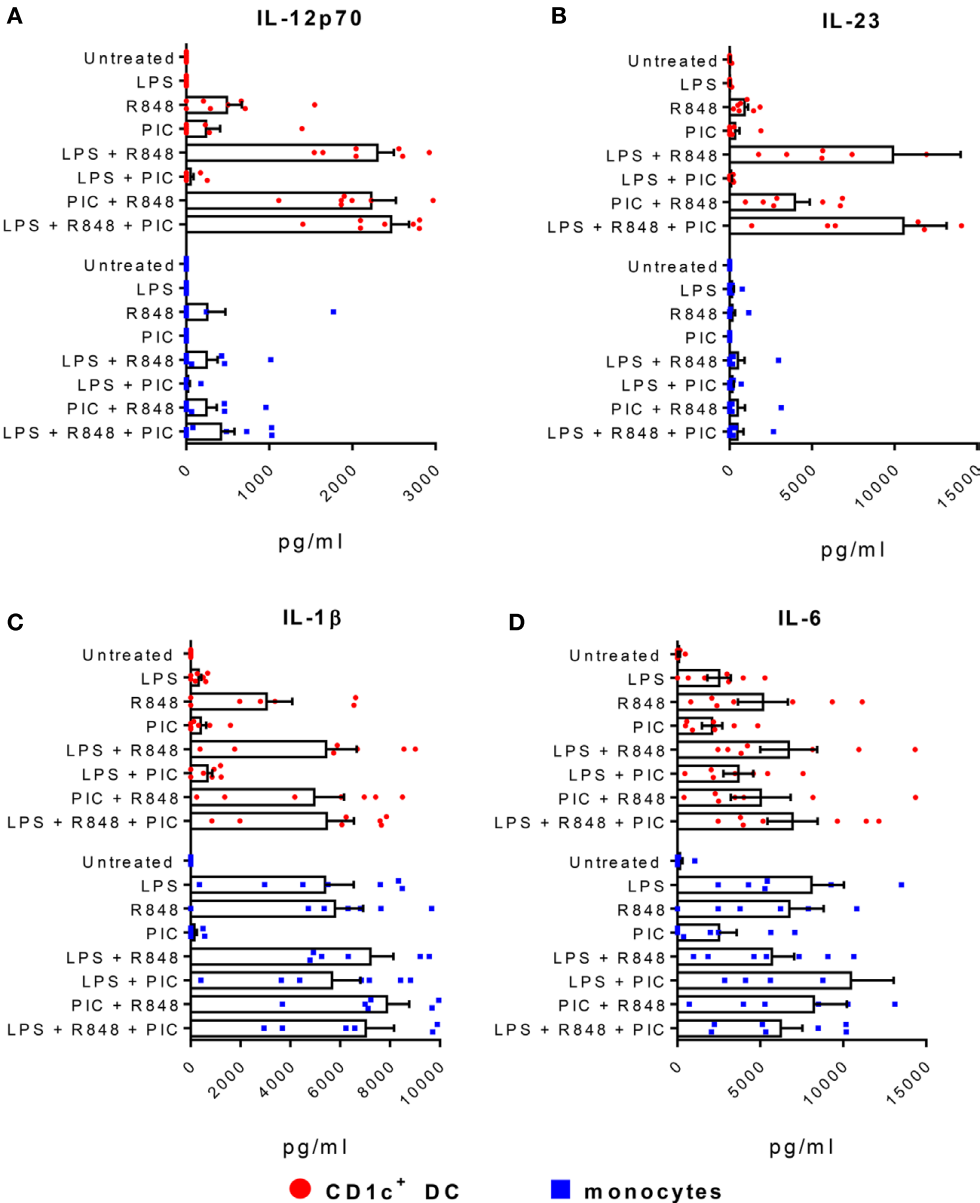
CD1c^+^ dendritic cells (DC) produce high levels of IL-23 and IL-12p70. Production of **(A)** IL-12p70, **(B)** IL-23, **(C)** IL-1β, and **(D)** IL-6 by CD1c^+^ DC (red) and monocytes (blue) following stimulation with LPS, R848, and/or poly I:C (PIC). Data points are eight individual donors with the mean + SEM shown. Cytokine levels are expressed as picogram per milliliter per 5 × 10^4^ cells.

CD1c^+^ DC produce high levels of IL-12p70 when stimulated with R848 combined with LPS or poly I:C (18, 20). Consistent with these findings, we observed high levels of IL-12p70 production when CD1c^+^ DC were stimulated with R848 + LPS or R848 + poly I:C (Figure 2A). The same conditions that induced IL-12p70 also stimulated high levels of IL-23 by CD1c^+^ DC, although the combination of R848 + poly I:C was less effective than R848 + LPS (Figure 2B). The combination of LPS + poly I:C did not induce detectable levels of IL-12p70 or IL-23, indicating a requirement for TLR7 and/or 8 signaling. The triple combination of R848 + LPS + poly I:C did not further augment IL-12p70 or IL-23 production over R848 + LPS. IL-1β and IL-6 were also produced by CD1c^+^ DC stimulated with R848 alone or in combination with LPS and/or poly I:C (Figures 2C,D). Despite high expression of TLR8 and TLR4 and robust secretion of IL-1β and IL-6 when stimulated with R848 or LPS alone or combined, blood CD14^+^ monocytes did not produce appreciable levels of either IL-12p70 or IL-23. Thus, R848 in synergy with either LPS or poly I:C concomitantly induces Th1 and Th17 polarizing cytokines IL-1β, IL-6, IL-12p70, and IL-23 by CD1c^+^ DC.

### CD1c^+^ DC Promote Th1 and Th17 Responses in Memory CD4^+^ T Cells

**Figure 3:**
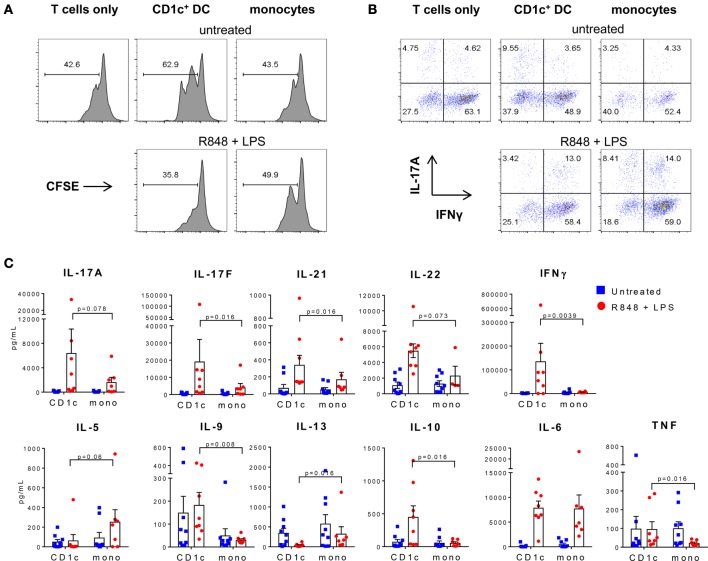
Activated CD1c^+^ dendritic cells (DC) induce Th1 and Th17 cytokines in memory CD4^+^ T cells. **(A)** Proliferation of memory CD4^+^ T cells cultured alone, or with CD1c^+^ DC or monocytes untreated or activated with R848 + LPS. Shown are the percentages of dividing cells measured by carboxyfluorescein succinimidyl ester (CFSE) dilution after 7 days of culture from one representative of three donors. **(B)** Production of interferon-γ and IL-17A from proliferating memory CD4^+^ T cells after restimulation with phorbol 12-myristate 13-acetate/ionomycin at day 7. **(C)** Cytokine production in the supernatants of memory CD4^+^ T cells after 7 days culture with CD1c^+^ DC or monocytes untreated or activated with R848 + LPS. Data points are seven to eight individual donors with the mean + SEM shown.

Since the cytokine profile of CD1c^+^ DC activated with R848 + LPS or poly I:C is consistent with a role in promoting both Th1 and Th17 responses, we examined the capacity of activated CD1c^+^ DC and monocytes to polarize autologous memory CD4^+^ T cells. As previously reported, memory CD4^+^ T cells proliferated in the presence of IL-7 and IL-15 and the presence of untreated CD1c^+^ DC enhanced proliferation to a greater extent than monocytes, although this varied between donors (Figure 3A) (31). Surprisingly, lower percentages of proliferating T cells were found after culture with CD1c^+^ DC activated with R848 + LPS (Figure 3A). Cultures stimulated with R848 + LPS-activated CD1c^+^ DC or monocytes contained similar proportions of CD4^+^ T cells capable of co-producing IFN-γ and IL-17A when re-activated with PMA and ionomycin compared with cultures stimulated with untreated CD1c^+^ DC or monocytes (Figure 3B). These data suggest that cultures stimulated with activated CD1c^+^ DC and monocytes contain memory CD4^+^ T cells that harbor the capacity to co-produce IL-17A and IFN-γ. However, the culture supernatants of memory CD4^+^ T cells stimulated with activated CD1c^+^ DC contained higher levels of IFN-γ, IL-17A, IL-17F, IL-21, and IL-22 compared with memory CD4^+^ T cells stimulated with activated monocytes, while supernatants from T cells cultured with unstimulated CD1c^+^ DC or monocytes contained low or undetectable levels of these cytokines (Figure 3C).

Memory CD4^+^ T cells cultured with activated CD1c^+^ DC also produced lower levels of Th2 polarizing cytokines IL-5 and IL-13 compared with cultures stimulated with similarly activated monocytes, and higher levels of IL-9, IL-10, and TNF (Figure 3C). IL-2 and IL-4 were low or below detectable levels in all cultures (not shown) and similar levels of IL-6 were detected in cultures stimulated with activated CD1c^+^ DC and monocytes (Figure 3C). These data demonstrate that although memory CD4^+^ T cells capable of producing IL-17A and IFN-γ were present in both activated CD1c^+^ DC and monocytes co-cultures after 7 days, activated CD1c^+^ DC promoted increased Th1 and Th17 and decreased Th2 effector function by CD4^+^ T cells that was characterized by higher levels of IFN-γ, IL-17A, IL-17F, IL-21, and IL-22 and lower IL-5 and IL-13 in the culture supernatants.

### CD1c^+^ DC Are the Main Human Blood DC Subset Producing IL-1β, IL-6, IL-12, and IL-23

**Figure 4:**
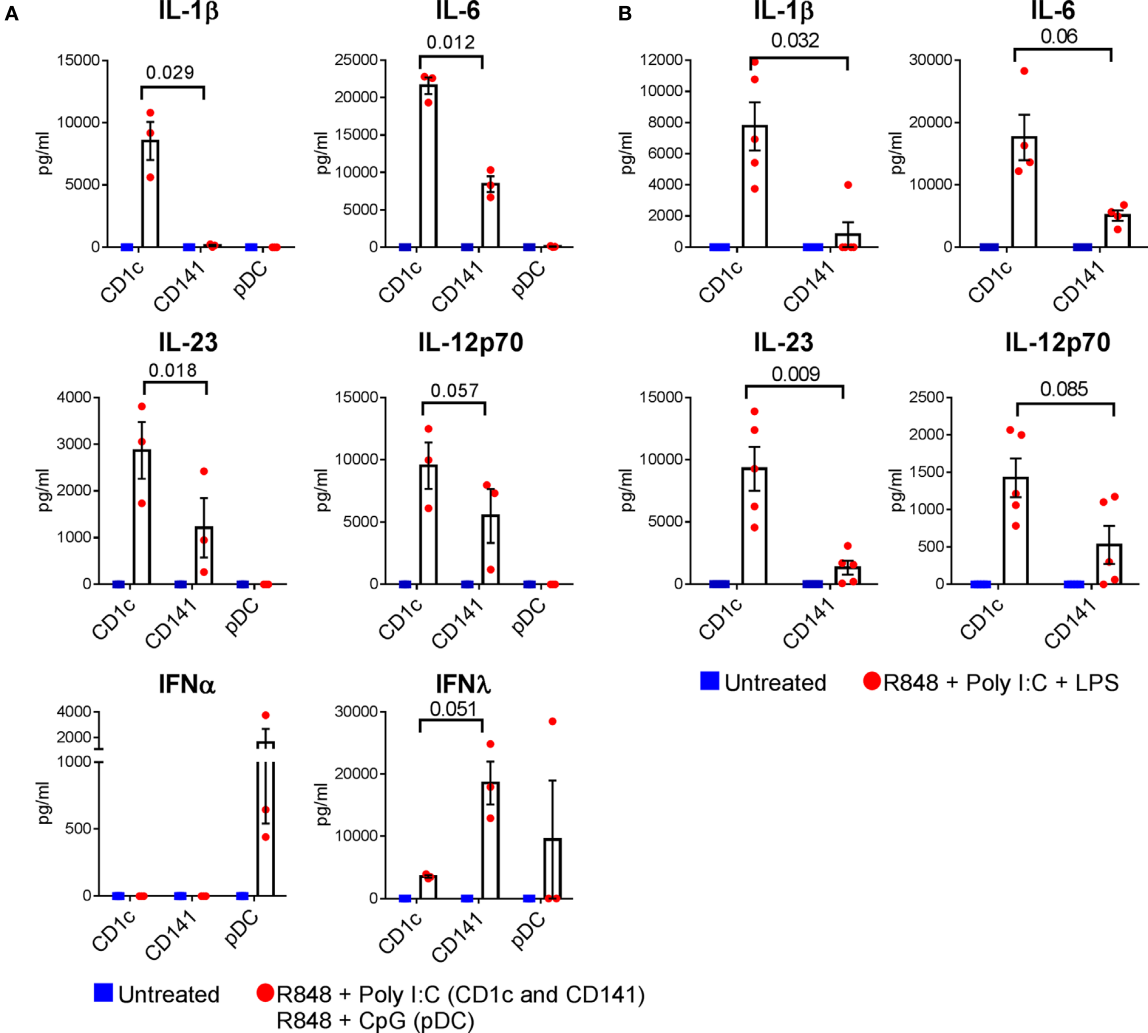
Cytokine production by optimally activated dendritic cells (DC) subsets. **(A)** Cytokine production by CD1c^+^ DC and CD141^+^ DC untreated or activated with R848 + poly I:C, and plasmacytoid DC activated with R848 + CpG. **(B)** Cytokine production by CD1c^+^ DC and CD141^+^ DC after activation with combined R848, poly I:C, and LPS. Data points are individual donors with mean ± SEM shown. Cytokine levels are expressed as picogram per milliliter per 5 × 10^4^ cells. *p* values are shown.

We next investigated whether the ability to produce IL-1β, IL-6, IL-12p70, and IL-23 and to activate memory CD4^+^ Th17/Th1 responses was unique to CD1c^+^ DC, or a more generalized feature of other human DC subsets. Because CD141^+^ DC and pDC do not express TLR4 we compared CD1c^+^ DC and CD141^+^ DC activated with R848 + poly I:C and pDC activated with R848 + CpG (Figure 4). CD1c^+^ DC were the only subset capable of producing detectable levels of IL-1β under these conditions (Figure 4A). Activated CD141^+^ DC produced IL-6 and IL-23 but the levels were significantly lower compared with CD1c^+^ DC derived from the same donors (Figure 4A). CD141^+^ DC also produced lower levels of IL-12p70 compared with CD1c^+^ DC in all donors tested, although this did not reach statistical significance. Activated pDC did not produce detectable levels of these cytokines. Under the same stimulatory conditions, pDC were the only subset to produce IFN-α, and CD141^+^ DC produced the highest levels of IFN-λ, confirming the functional integrity of these cells. The superior capacity of CD1c^+^ DC to produce IL-1β, IL-6, and IL-23 compared with CD141^+^ DC was also apparent when both cell types were activated with the triple combination of R848 + poly I:C + LPS (Figure 4B), although the levels of IL-6 and IL-12p70 did not reach statistical significance. Thus, CD1c^+^ DC are superior to CD141^+^ DC and pDC in their capacity to produce IL-1β, IL-6, and IL-23 in response to combinatorial stimuli containing R848.

**Figure 5:**
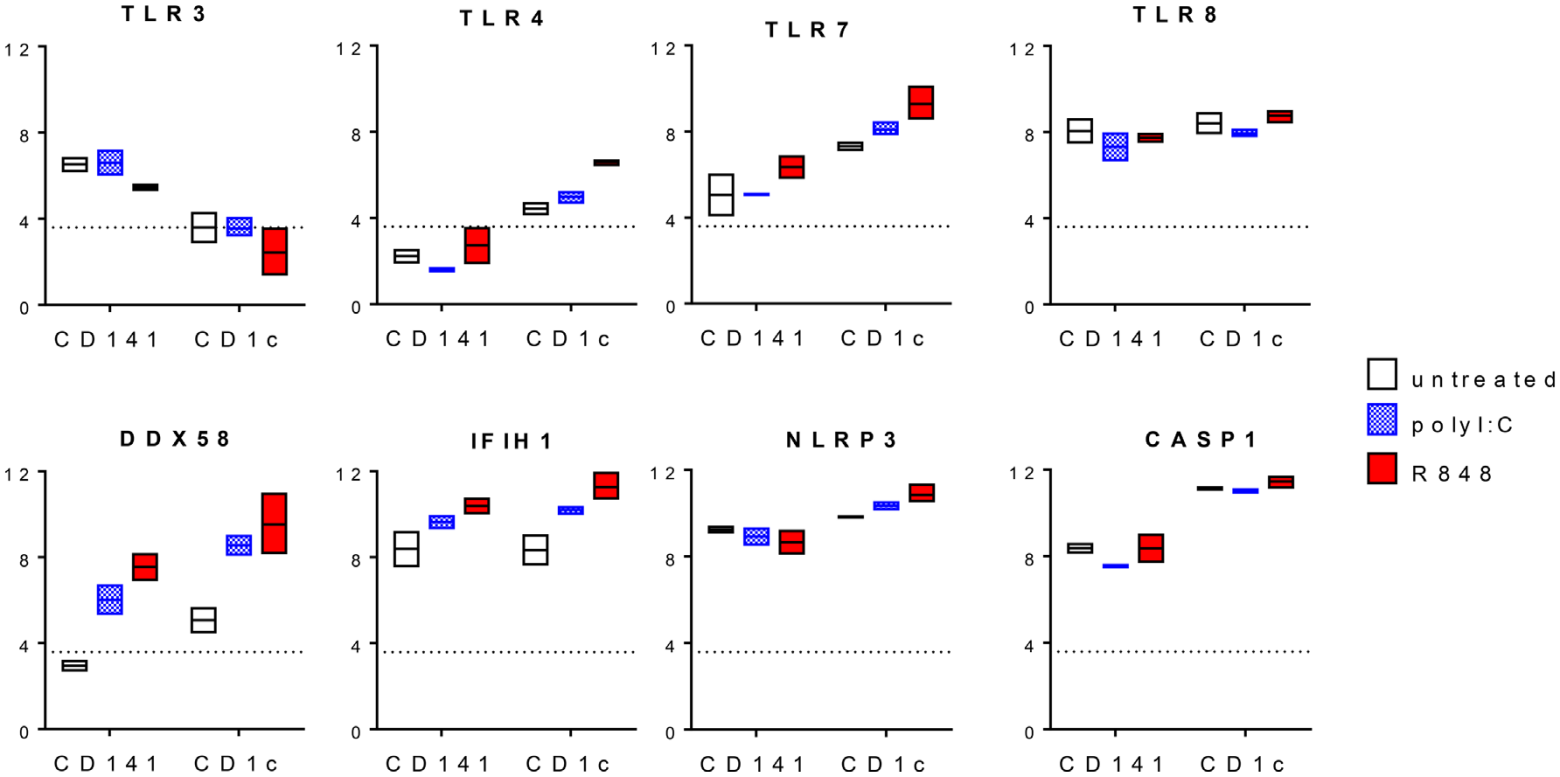
Changes in pattern recognition receptor expression after activation of CD1c^+^ dendritic cells (DC) and CD141^+^ DC. Normalized log 2 gene expression was calculated from data for CD1c^+^ DC and CD141^+^ DC untreated or activated with poly I:C or R848 *in vivo* obtained from GEO GSE99666. Boxes represent 25th–75th percentiles ± minimum and maximum values with line at the median from two to three individual donors. The expression threshold determined by the bi-modal distribution of detected versus non-detected probes was 3.6 (dotted line).

To provide some insights into the mechanism by which CD1c^+^ DC produced higher levels of Th1 and Th17 polarizing cytokines compared with CD141^+^ DC, we examined changes in PRR expression after activation (Figure 5). For this, we took advantage of a dataset of human CD1c^+^ DC and CD141^+^ DC activated with poly I:C or R848 *in vivo*. *TLR3* gene expression levels were unchanged after poly I:C activation and downregulated after R848 stimulation by CD141^+^ DC while *TLR8* expression did not markedly change (Figure 5). R848 induced expression of *TLR4* in CD1c^+^ DC and higher levels of TLR7 in CD1c^+^ DC compared with CD141^+^ DC. RIG-1/MDA-5 pathway genes *DDX58* and *IFIH1* were upregulated by both DC subsets after activation, with the highest expression seen in CD1c^+^ DC activated with R848. Thus, upregulation of RIG-I pathway genes rather than *TLR3* following DC activation may explain the increased responsiveness of CD1c^+^ DC and to a lesser extent, CD141^+^ DC, after activation with R848 + poly I:C. IL-1β production is mediated by inflammasomes, and in particular the NLRP3 inflammasome, which can be directly triggered by R848 (35). NLRP3 stimulation activates caspase-1 that mediates cleavage of the inactive IL-1β precursor to release active IL-1β. Compared with CD141^+^ DC, CD1c^+^ DC expressed higher levels of *NLRP3* and *CASP1* mRNA which were further upregulated with R848 activation, consistent with production of IL-1β by CD1c^+^ DC (Figure 5).

### CD1c^+^ DC But Not CD141^+^ DC or pDC Promote Th1 and Th17 Responses in Memory CD4^+^ T Cells

**Figure 6:**
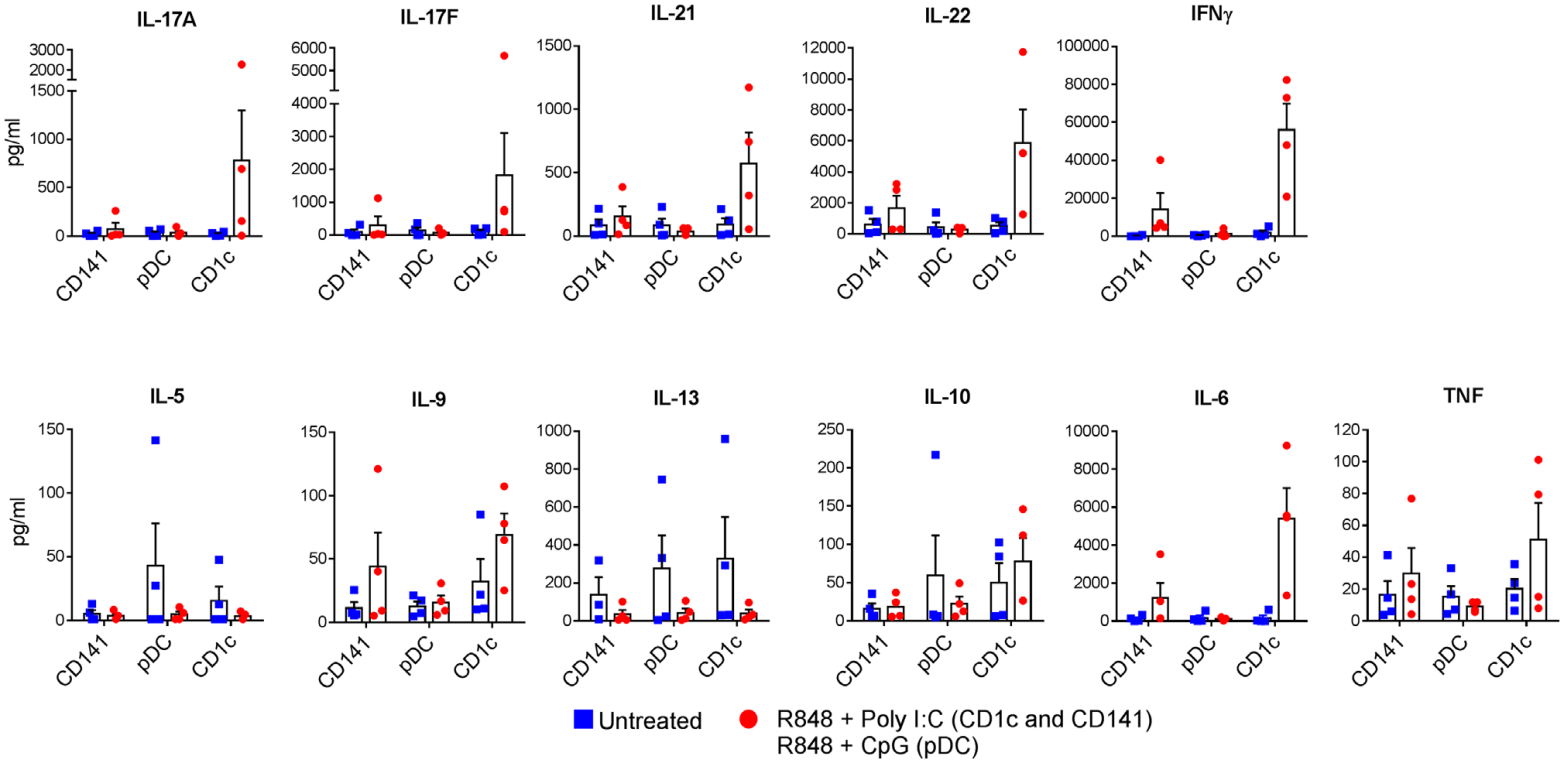
Production of Th1 and Th17 cytokines by memory CD4^+^ T cells activated with human dendritic cells (DC) subsets. Memory CD4^+^ T cells were cultured with CD1c^+^ DC or CD141^+^ DC activated with R848 + poly I:C, or plasmacytoid DC activated with R848 + CpG and cytokines measured in the culture supernatants after 7 days. Data points are four individual donors with mean + SEM shown.

The ability of activated CD1c^+^ DC to produce IL-1β and higher levels of IL-6, IL-12p70, and IL-23 compared with CD141^+^ DC and pDC suggested that CD1c^+^ DC would be the main DC subset to drive Th17/Th1 effector function in memory CD4^+^ T cells. We therefore compared the ability of CD1c^+^ DC and CD141^+^ DC activated with R848 + poly I:C, and pDC activated with R848 + CpG, to induce effector cytokine production in memory CD4^+^ T cells. Consistent with their cytokine secretion profiles, activated CD1c^+^ DC induced substantially higher production of IL-17A, IL-17F, IL-21, IL-22, IFN-γ, and IL-6 by memory CD4^+^ T cells compared with activated CD141^+^ DC or pDC in all four donors tested, although statistical significance was not reached (Figure 6). Secretion of other cytokines including IL-2, IL-4, IL-5, IL-9, IL-10, IL-13, and TNF was low or below detection and not noticeably different in the cultures stimulated by different DC subsets. Collectively these data suggest that CD1c^+^ DC are the main human blood DC subset responsible for inducing memory CD4^+^ Th1 and Th17 cells under these conditions.

## Discussion

This study investigated the ability of human blood DC subsets to promote Th1 and Th17 responses in memory CD4^+^ T cells. In mice, Th1 and Th17 responses are regulated by different cDC subsets, with cDC1 being the main producers of IL-12p70 and inducers of Th1 responses, while cDC2 produce IL-23 to promote Th17 responses. Our data showed that human CD1c^+^ DC, which aligns with mouse cDC2, produce both IL-12p70 and IL-23 in response to R848 combined with LPS or poly I:C. Although CD141^+^ DC, which align with mouse cDC1, were also capable of producing IL-12p70 and IL-23, they were not as proficient as CD1c^+^ DC. The unique combination of IL-1β, IL-6, IL-12p70, and IL-23 produced by CD1c^+^ DC activated with R848 + LPS or R848 + poly I:C endowed them with the capacity to promote both Th1 and Th17 effector function in memory CD4^+^ T that was characterized by high CD4^+^ T cell production of IFN-γ, IL-17A, IL-17F, IL-21, and IL-22 and low production of Th2 cytokines IL-5 and IL-13. These findings identify CD1c^+^ DC as the main human blood DC subset to promote memory CD4^+^ Th1 and Th17 responses.

The apparent discrepancy between mouse and human cDC subsets may be explained by interspecies differences in TLR expression and responsiveness to TLR ligands. In humans, R848 stimulates both TLR7 and TLR8 independently and possibly in synergy (36, 37). Although CD1c^+^ DC and CD141^+^ DC express similar levels of TLR8 mRNA, CD1c^+^ DC express higher levels of TLR7 mRNA that is further upregulated by stimulation with R848, and to a lesser degree, poly I:C. This is not dissimilar to the mouse, where both cDC1 and cDC2 express TLR8, but only cDC2 express TLR7 (38). However, mouse TLR8 does not recognize the same ssRNA ligands as human TLR8 and is not responsive to R848 (36, 39). Indeed, stimulation of mouse DC subsets with R848 results in expression of the shared IL-12/IL-23 p40 subunit by cDC2 but not cDC1 (38). Thus, stimulation of both TLR7 and TLR8 may explain, at least in part, why CD1c^+^ DC are more responsive to R848 compared with CD141^+^ DC. However, it remains to be seen whether mouse cDC2 also acquire a similar ability to produce IL-12p70 and IL-23 if appropriately stimulated with ligands specific for both mouse TLR7 and 8. RIG-1/MDA-5 pathway genes *DDX58* and *IFIH1*were also more highly expressed by activated CD1c^+^ DC. This is consistent with expression of RIG-I and MDA-5 by their cDC2 mouse counterparts and combined with increased TLR7 suggests a common role for this DC lineage in responses to ssRNA viruses (40).

Our data concurred with previous studies showing tight regulation of IL-12p70 that requires combinatorial TLR stimulation for high level production, particularly when CD1c^+^ DC are activated with R848 combined with either LPS or poly I:C (17–20). Similar findings have been reported for human MoDC and mouse bone marrow-derived DC demonstrating that high IL-12p70 production under these conditions is not unique to CD1c^+^ DC (25, 41). We found that IL-23 was also secreted in high levels by CD1c^+^ DC in response to combinatorial stimulation of R848 with LPS and to a lesser extent with poly I:C. The synergistic effects of LPS on IL-12p70 production have been proposed to occur *via* TLR4 activation of IRF3, leading to Type I IFN production, that is, required for optimal IL-12p70 (41). We demonstrated rapid upregulation of TLR4 mRNA on CD1c^+^ DC but not CD141^+^ DC upon R848 stimulation, thereby allowing CD1c^+^ DC to be receptive to LPS stimulation. Thus, IL-12p70 production by CD1c^+^ DC in the presence of R848 + LPS is most likely mediated by activation of the NF-κB pathway *via* TLRs 4,7, and 8, and potentially enhanced by Type I IFN *via* TLR4 activation of IRF3. A similar mechanism may also explain the enhanced production of IL-23 and IL-6 under these conditions. In contrast to humans, both mouse cDC1 and cDC2 express TLR4 but whether TLR7/8 and TLR4 synergize for IL-12p70 and IL-23 in specific mouse cDC subsets remains to be tested.

The synergistic effects of poly I:C with R848 on IL-12p70 production were also reported to be mediated by Type I IFN induced by TLR3 activation of IRF3 in mouse bone marrow-derived DC and human MoDC (41). However, this is unlikely to be the case for CD1c^+^ DC since TLR3 mRNA expression on these DC remained barely detectable even after R848 activation. Furthermore, CD141^+^ DC, which maintain high levels of TLR3 expression after activation, produced much lower levels of IL-12p70 and IL-23 compared with CD1c^+^ DC in response to poly I:C + R848. Interestingly, *DDX58* and *IFIH1* expression increased on both DC subsets after activation, with the highest expression found on R848 activated CD1c^+^ DC. Activation of the RIG-I/MDA-5 pathway, which also induces Type I IFN, may therefore be the pathway by which poly I:C synergizes with R848 on CD1c^+^ DC.

In addition to triggering TLR7 and 8, R848 directly activates the NLRP3 inflammasome pathway that leads to secretion of bioactive IL-1β (35). Our data showed that CD1c^+^ DC expressed higher levels of inflammasome pathway genes, *NLRP3* and *CASP1*, compared with CD141^+^ DC, particularly after R848 activation. Consistent with this, CD1c^+^ DC secreted IL-1β in response to R848 and this was not markedly increased when combined with LPS or poly I:C. Our data showing production of IL-1β and IL-6 but not IL-23 or IL-12p70 by monocytes is consistent with a previous report (17). We further demonstrated that CD141^+^ DC do not secrete IL-1β and produce lower levels of IL-6 and IL-23 compared with CD1c^+^ DC, while pDC did not produce detectable levels of any of these cytokines. Thus, CD1c^+^ DC appear unique in their capacity to produce the combination of IL-1β, IL-6, IL-12p70, and IL-23 in response to R848 + LPS or R848 + poly I:C.

The high levels of IL-12p70 produced following activation of MoDC or CD1c^+^ DC with R848 + LPS primes naïve CD4^+^ and CD8^+^ T cells for powerful Th1 responses and IFN-γ production (17, 20, 25). Our data showed that R848 + LPS and R848 + poly I:C activated CD1c^+^ DC also induced memory CD4^+^ T cells to produce high levels of IFN-γ. We demonstrated decreased Th2 cytokines IL-5 and IL-13 induced by CD1c^+^ DC after activation, which may further contribute to increased Th1 phenotype. However, we also showed under the same Th1-biased conditions that CD1c^+^ DC, but not monocytes, CD141^+^ DC or pDC, are strong inducers of the Th17 effector cytokines IL-17A, IL-17F, IL-21, and IL-22 in memory CD4^+^ T cells. This is consistent with the ability of CD1c^+^ DC to produce known Th17 promoting cytokines IL-1β, IL-6, and IL-23 under these conditions, although the precise contributions of these cytokines in this model are yet to be elucidated. This differs to the priming of naïve CD4^+^ T cells, where Th17 responses are inhibited in the presence of IL-12p70 (17, 25). Thus, CD1c^+^ DC have the ability to simultaneously promote Th1 and Th17 responses in memory CD4^+^ T cells, even under Th1-biased conditions.

Th17 responses, and in particular IL-17^+^/IFN-γ^+^ double positive cells, are important in the pathogenesis of many human inflammatory conditions including rheumatoid arthritis, psoriasis, and inflammatory bowel disease (26). Although IL-1β is required for inducing IL-17^+^/IFN-γ^+^ cells (29, 42), IL-12p70 has been shown to reprogram memory Th17 cells to co-produce IFN-γ and classical Th17 cytokines (11, 29, 43). Memory CD4^+^ T cell cultures contained similar proportions of cells with the capacity to co-produce IFN-γ and IL-17, whether stimulated with activated monocytes or activated CD1c^+^ DC. Given that only activated CD1c^+^ DC produced IL-12 while both activated CD1c^+^ DC and monocytes produce IL-1β, this observation would suggest that the IL-17^+^/IFN-γ^+^ phenotype is induced by IL-1β rather than IL-12p70 in our study. IL-12p70 and IL-23 production by CD1c^+^ DC therefore appears most likely to enhance the level of effector cytokine production by Th1 and Th17 memory CD4^+^ T cells rather than directing expansion of IL-17^+^/IFN-γ^+^ cells. However, the precise role of human DC in the activation of memory CD4^+^ T cell subsets will require further evaluation following stimulation with a broader range of known Th1, Th17, Th1/17, and Th2 and specific activation of purified memory CD4^+^ Th subsets.

Combinatorial TLR stimulation, and in particular R848 + poly I:C, are considered as promising vaccines adjuvants owing to the ability to induce powerful Th1 responses (44). They have been safely administered in phase I clinical trials for cancer patients (45). The role of Th17 responses in tumor immune responses is controversial (46) and the activation of effector memory Th17 cells *via* combinatorial TLR stimulation of CD1c^+^ DC now requires consideration in this context (46). Aberrant TLR signaling has also been implicated in driving autoimmune inflammation (47). Our data suggest a possible role for CD1c^+^ DC in contributing to this process. The DC present in human inflammatory tissues are genetically more similar to MoDC than CD1c^+^ DC and have also been shown to induce IL-17 production by memory CD4^+^ T cells (21, 22). Unlike CD1c^+^ DC, MoDC do not produce large amounts of IL-1β after activation with R848 + LPS (17, 19). However, after stimulation with a TLR2 ligand, MoDC can also produce large amounts of IL-1β and IL-23 and facilitate IL-17 production by memory CD4^+^ T cells in an IL-1β and IL-23-dependent manner (23). MoDC and CD1c^+^ DC arise from separate precursors but share many overlapping phenotypic and functional features, including promotion of memory Th1 and Th17 CD4^+^ T cells. Whether they play specific roles or there is functional redundancy of these DC in different contexts of autoimmune inflammation is therefore likely to be a complex and interesting area for future investigation.

Aside from systemic autoimmunity, other conditions associated with increased intestinal permeability provide opportunities for TLR stimuli to enter the circulation and directly encounter CD1c^+^ DC and memory T cells. Blood CD1c^+^ DC have recently been segregated into two subpopulations, a non-inflammatory subset defined by expression of *FCER1A, CLEC10A*, and *FCGR2*, and an inflammatory subset defined by expression of *CD36* and *CD163* (48). Which of these subsets produce Th1 and Th17 polarizing cytokines will be an important area for future investigation. Likewise it remains to be seen whether these functions are conserved in CD1c^+^ DC in other tissues. CD1c^+^ DC in blood and lymphoid tissues are closely related to each other whereas CD1c^+^ DC in non-lymphoid tissue are heavily influenced by their microenvironment and may therefore function differently to their blood counterparts (11). Further consideration will need to be taken into the distribution and function of memory T cell subsets in different organs and the influence of different DC subsets on them at these sites (49).

## Ethics Statement

Whole blood and leukapheresis products from healthy volunteers were obtained for this study following approval from Mater Health Services Human Research Ethics Committee and with informed consent.

## Author Contributions

IR, W-HM, FP, and YM designed and performed experiments and analyzed data. TK, and RB provided input into project design, data analysis, and interpretation of results, KR conceptualized the project and experimental design, analyzed and interpreted data, and wrote the manuscript.

## Conflict of Interest Statement

The authors declare that the research was conducted in the absence of any commercial or financial relationships that could be construed as a potential conflict of interest.
