# Supplementary figures and images for: Human Blood CD1c+ Dendritic Cells Promote Th1 and Th17 Effector Function in Memory CD4+ T Cells
Source: Front Immunol. 2017 Aug 17;8:971. doi: 10.3389/fimmu.2017.00971 (PMC5572390; doi:10.3389/fimmu.2017.00971)

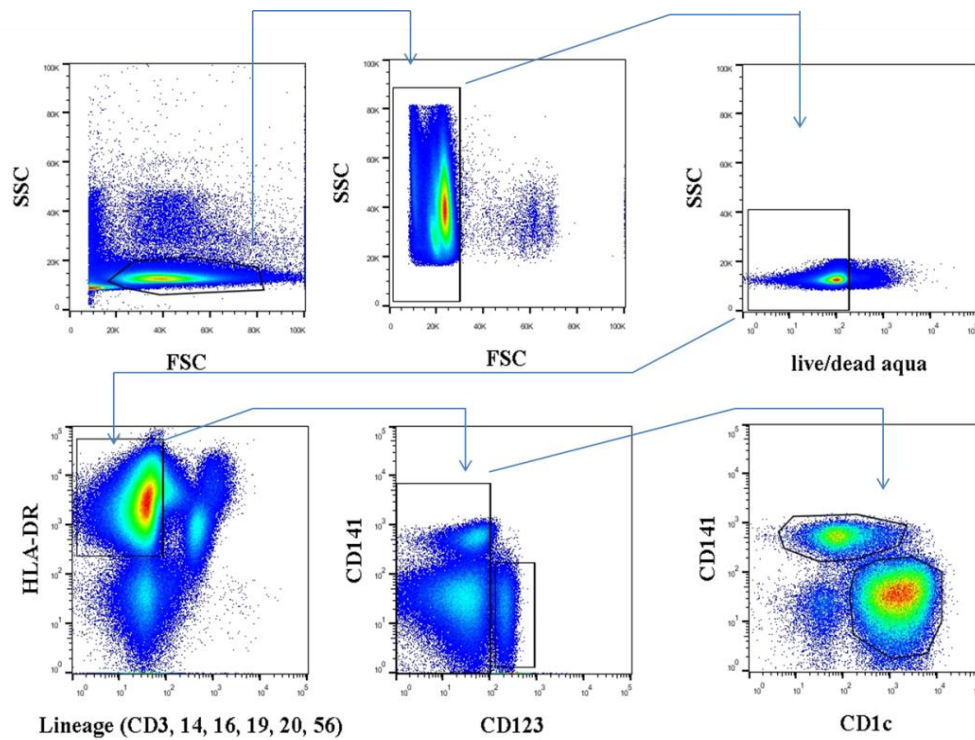

Suppl Fig 1: Gating strategy for flow cytometry sorting of DC subsets

Supplement: Supplementary file 1 [file Image_1.PDF]
